# Change in abundance of three phytophagous mite species (Acari: Eriophyidae, Tetranychidae) on quackgrass in the presence of choke disease

**DOI:** 10.1007/s10493-016-0060-3

**Published:** 2016-07-07

**Authors:** Brian G. Rector, Marcin Czarnoleski, Anna Skoracka, Marlena Lembicz

**Affiliations:** 1 USDA-ARS, Great Basin Rangelands Research Unit, Reno, NV USA; 2 Institute of Environmental Sciences, Jagiellonian University, Gronostajowa 7, 30-387 Kraków, Poland; 3 Department of Animal Taxonomy and Ecology & Population Ecology Lab, Faculty of Biology, Adam Mickiewicz University, Umultowska 89, 61-614 Poznań, Poland; 4 Department of Plant Taxonomy, Faculty of Biology, Adam Mickiewicz University, Umultowska 89, 61-614 Poznań, Poland

**Keywords:** Endophyte, Epichloë, Herbivory, Interspecific interactions, Poaceae, Symbiosis

## Abstract

Phytophagous mites and endophytic fungi may interact when sharing a host plant, potentially influencing one another’s growth or population dynamics; however, interactions between them are poorly known and remain largely unexplored. In this study, quantitative associations between three species of phytophagous mites and the endophytic fungus *Epichloë bromicola* Leuchtm. & Schardl (Clavicipitaceae, Ascomycotina) on quackgrass, *Elymus repens* (L.) Gould are reported. The mites’ abundance was assessed on field-collected grass shoots that were either exhibiting choke disease symptoms or without the fungus. Overall, the abundance of *Tetranychus urticae* and *Aculodes mckenziei* was significantly lower on quackgrass plants infected by *E. bromicola* compared to plants without the fungus. Conversely, populations of *Abacarus hystrix* were significantly larger on plants colonised by the fungus than on uninfected plants. Thus, the presence of this endophytic fungus may have divergent effects on different phytophagous mite species although the basis of these effects is not yet known.

## Introduction

Spider mites (Tetranychidae) and eriophyoid mites (Eriophyoidea) are considered the most economically important taxa of all plant-feeding mites (Hoy 2011). Tetranychids typically have wide host ranges (Bolland et al. 1998), whereas the majority of eriophyoids are highly host specific (Skoracka et al. 2010). Tetranychidae are considered major plant pests worldwide, attacking food crops, trees, and ornamentals, causing serious yield losses. The most notorious pest species is the two-spotted spider mite, *Tetranychus urticae* Koch., which has a worldwide distribution and a wide host range (Bolland et al. 1998; Migeon and Dorkeld 2006–2011; Hoy 2011). Many of eriophyoids are also significant crop pests, some of which represent quarantine threats to numerous countries due to their direct feeding damage as well as transmission of plant diseases by some species (e.g. Duso et al. 2010; Navia et al. 2010).

**Fig. 1:**
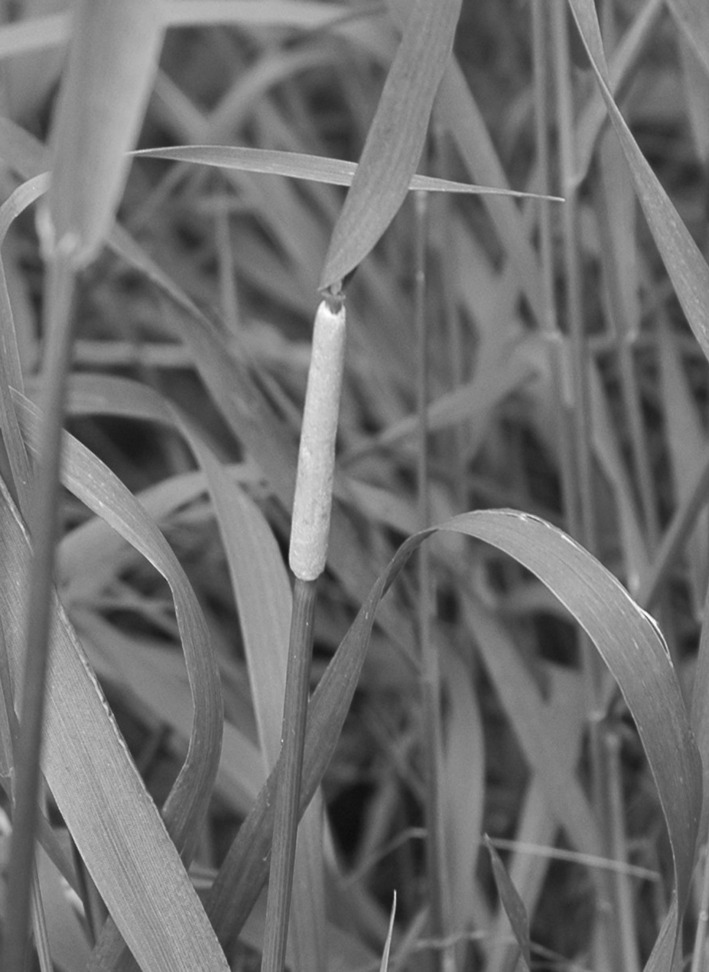
Fertilized stromata of *Epichloё bromicola* on infected stems of quackgrass (*Elymus repens*). phot. M. Lembicz

Endophytic fungi are virtually ubiquitous symbionts living within plant tissues (Saikkonen et al. 2004; Cheplick and Faeth 2009; Rodriguez et al. 2009) that may protect their host plants either directly, e.g. through production of alkaloids that make them toxic or less palatable to herbivores (e.g. Bacon 1995; Elliot et al. 2000; Czarnoleski et al. 2012; García Parisi et al. 2014), or indirectly, e.g. by enhancing detection of mite (Schausberger et al. 2012) and even mammalian herbivores (Huitu et al. 2008) by predators. Alkaloids associated with the presence of endophytic fungi in grasses may reduce damage by herbivorous insects (Potter et al. 2008), via reduced feeding, oviposition (Rowan et al. 1990) or overall insect performance (Breen 1994; Clay and Schardl 2002). Not all endophytic fungi are known to benefit their hosts. For example, many epichloae (i.e. *Epichloë* and *Neotyphodium* species; Clavicipitaceae) are endophytes that produce no symptoms and are transmitted vertically through host lineages (i.e. via host seeds) without reproducing sexually themselves (Brem and Leuchtmann 2003; Schardl et al. 2013). However, some *Epichloë* spp., cause “choke disease”, whereby they produce fruiting bodies (stromata) containing sexually reproductive spores and prevent flower and seed development in their hosts (Fig. 1). This disease is a significant threat to some economically important grasses (Western and Cavett 1959; Siegel et al. 1987; Brem and Leuchtmann 2003).

Although we know much about interactions of plant-symbiotic fungi with insects (e.g. Rowan et al. 1990; Breen 1994; Elliot et al. 2000; Clay and Schardl 2002; Potter et al. 2008; García Parisi et al. 2014), effects of these symbionts on mite herbivores have largely focused on interactions between root-associated microorganisms and the cosmopolitan pest, *T. urticae* (reviewed by Hoffmann and Schausberger 2012). For example, Zhang et al. (2012) demonstrated that the endophyte *Neotyphodium gansuense* Li & Nan, which is associated with drunken horse grass, *Achnatherum inebrians* (Hance) Keng, reduced both feeding and survival of the spider mite *Tetranychus urticae* Koch.

The endophyte *Epichloë bromicola* Leuchtm. & Schardl has been recorded from numerous grass species and is known to produce choke disease in several of them (Brem and Leuchtmann 2003; Song and Nan 2015). The purpose of this study was to report quantitative and descriptive associations between choke-producing *Ep. bromicola* and the phytophagous mites *Abacarus hystrix* (Nal.), *Aculodes mckenziei* (Keif.) (both Eriophyidae) and *T. urticae* (Tetranychidae) on their shared host quackgrass, *Elymus repens* (L.) Gould.

## Materials and methods

The study plant, quackgrass (*El. repens*), is native to Europe and Asia. In Poland it is a common weed of field crops (Zając and Zając 2001). In May 2008, signs of the fungus *Ep. bromicola* were observed on the shoots of *El.repens* at two localities in Poland (Lembicz et al. 2010). In 2011, shoots of *El. repens*, with and without visible stromata of *Ep. bromicola* were collected from distinct clusters of *El. repens* shoots along four transects at three sites in Poland (Table 1). Each transect was 15 m long and 1 m wide. One shoot with and one shoot without stromata were collected from within each 1 × 1 m square along the length of each transect. If there was no *El. repens* in a given square, no shoots were collected in that square. Plant shoots were placed separately in plastic bags inside a cooler. Each collected shoot, whether with or without visible signs of choke disease, was checked for the presence of the endophytic form of the fungus, which is evident from hyphae in intercellular spaces that stain dark blue with aniline blue dye. Specimens were analysed using a light microscope. The fungus on collected shoots was identified as *Ep. bromicola*, based on matching of nucleotide sequences of *tubB* introns (GenBank Accession No. DQ267692). Molecular identification followed the procedures of Brem and Leuchtmann (2003) and Lembicz et al. (2010). Each shoot designated as being without stromata was further tested for the presence of the asexual, asymptomatic stage of the fungus with aniline blue staining of leaf sheath epidermis and observations under a light microscope.

**Table 1:** Characteristics of the sampled sites. Please note that the raw data on the abundance of mites are not adjusted by differences in plant size

Site name	GPS coordinates	*Epichloë* presence	No. of shoots sampled	No. of shoots with			Mean no. of mites per shoot		
				ABH	ACM	TEU	ABH	ACM	TEU
Dulsk 1 (D1)	N 52° 45′ 23.42″	No	10	9	1	1	13.7	0.8	1.3
	E 18° 21′ 22.03″	Yes	11	11	1	4	42.2	0.1	1.1
Dulsk 2 (D2)	N 52° 45′11.32″	No	11	9	4	11	17.6	22.8	184.9
	E 18° 19′35.27″	Yes	12	5	0	6	2.5	0	32.6
Jacewo (J)	N 52° 48′ 02.88″	No	13	12	3	5	9.6	2.6	2.1
	E 18° 17′ 50.78″	Yes	12	8	0	7	5.7	0	4.5
Pakość (P)	N 52° 48′ 06.23″	No	11	7	0	2	36.5	0	1.3
	E 18° 05′ 07.76″	Yes	17	13	0	6	32.0	0	9.8

*ABH* *Abacarus hystrix*, *ACM* *Aculodes mackenziei*, *TEU* *Tetranychus urticae*

For each collected shoot, the length and the number of leaves were recorded and these measures were used to estimate the relative sizes of the plants (see below). The shoots were also examined under a stereo-microscope (Olympus SZX16) to detect mites. Mites were counted and were subsequently mounted on slides in Heinze medium (Heinze 1952; de Lillo et al. 2010). Mites were then identified to species (Manson 1967; Baker and Tuttle 1994; Skoracka 2004, 2009) using an Olympus BX41 phase-contrast light microscope.

### Data analysis

Before statistical analysis the data on shoot length and number of leaves per shoot were log10-transformed and a Principal Components Analysis was performed on these values. We used the scores of the first principal component as our integrated measure of plant size.

To examine links between endophyte presence and abundance of mites on quackgrass, a Generalized Linear Model (GLM) was employed for the number of mites on plant shoots, assuming Poisson distribution and a log-link function. A separate analysis was performed for each mite species and each model included infection status and study location as grouping variables. Larger plants were expected to harbor larger numbers of mites. To eliminate this bias caused by a simple scaling effect, our models considered the index of plant size as a numerical covariate. Thus, comparisons between our study groups were made for plants adjusted to the mean plant size. The analyses were performed using Statistica 10 (StatSoft, Poland).

## Results

Three species of mites were collected from the *El. repens* shoots: the polyphagous spider mite *T. urticae* (Tetranychidae) and two plant mites that are commonly found on grasses, *Ab. hystrix* and *Ac. mackenziei* (both Eriophyidae). The most numerous species was *Ab. hystrix*, followed by *T. urticae*, whereas *Ac. mackenziei* was found in very small numbers (Table 1).

The PCA of leaf number and shoot length showed that both parameters were highly positively correlated and they formed the first principal component (i.e. our index of plant size in subsequent analyses), explaining 92 % of the variation in the data (loadings of both parameters were equal to 0.96).

**Fig. 2:**
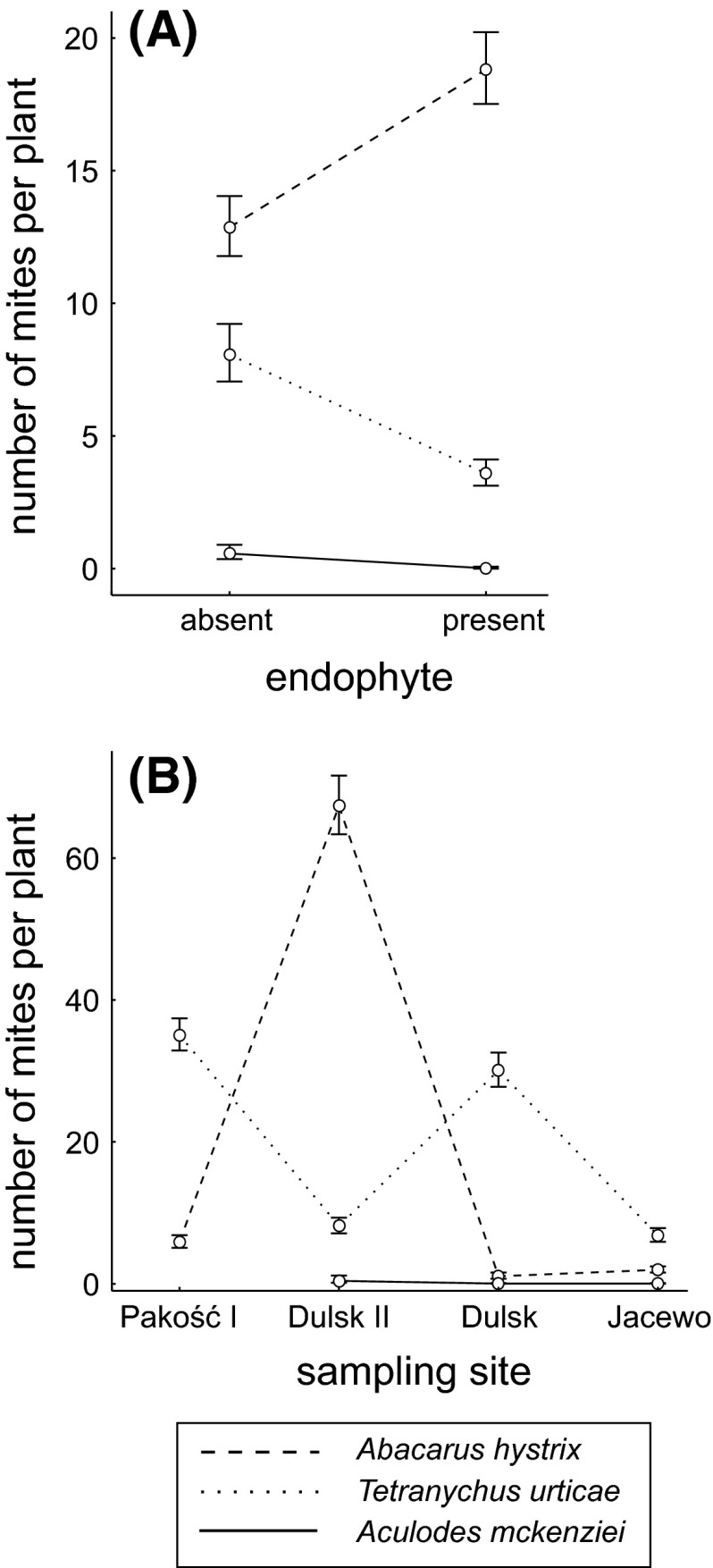
**a** Number of mites belonging to three species occupying the grass *Elymus repens* either with or without the endophytic fungus *Epichloё bromicola*. **b** Difference in the number of mites on plants at different study sites. Because the number of mites was positively related to the size of plants, the *graphs* show mean numbers of mites with confidence intervals, modeled for an average-size plant (see Table 2)

As predicted, results of the GLM (Table 2) indicated that larger plants harboured more mites. After accounting for this scaling effect, we found that *T. urticae* and *Ac. mckenziei* were most abundant on endophyte-free plants, in contrast to *Ab. hystrix*, which was found in the highest numbers on endophyte-infected plants (Fig. 2a). The abundance of mites also differed between the study sites; *T. urticae* and *Ac. mckenziei* were most abundant at the Dulsk 2 (D2) site; whereas *Ab. hystrix* reached the highest numbers in Pakość (P) and both Dulsk (D1 and D2) sites (Fig. 2b).

**Table 2:** Results of generalized linear models (likelihood type 3 test) examining the effects of endophytic infection and study site (grouping predictors), and plant size (numerical predictor-covariate) on the number of mites infesting plants. Data on each mite species were analyzed with a separate model. Please note that the model adjusted comparisons between groups for a mean plant size

	df	Log-likelihood	Chi-square	*p*
*Tetranychus urticae*				
Study site	3	−4340.48	4667.772	0.000001
Endophyte presence	1	−2117.72	222.255	0.000001
Plant size index	1	−2136.41	259.638	0.000001
*Abacarus hystrix*				
Study site	3	−2239.76	833.3133	0.000001
Endophyte presence	1	−1845.89	45.5701	0.000001
Plant size index	1	−1909.06	171.9009	0.000001
*Aculodes mckenziei*				
Study site	2	−527.357	342.4036	0.000001
Endophyte presence	1	−389.633	66.9559	0.000001
Plant size index	1	−461.451	210.5917	0.000001

## Discussion

Our comparative study revealed a complex pattern in the co-occurrence of phytophagous mites and an endophytic fungus on quackgrass. The mites *T. urticae* and *Ac. mckenziei* were most abundant on endophyte-free plants, which was consistent with our expectations that the presence of endophyte can result in lower pressure of phytohagous mites on grass. At the same time we found more mites of *Ab. hystrix* on endophyte-infected plants, which was contrary to our hypothesis. This suggests that the presence of an endophyte, in this case the sexual form of *E. bromicola* may have contrasting effects on different species of phytophagous mites feeding on the diseased host plant. Such divergent effects have also been observed in phytophagous insects of different orders and differing host acceptance traits (i.e. generalist vs. specialist) in response to host infection by fungal endophytes (Gange et al. 2012). We also consider that the overall interaction between mites and the endophyte can be much more complex, as it involves direct interactions between the three species of mites. Such interactions could explain why the increased abundance of *T. urticae* and *Ac. mckenziei* on endophyte-free plants coincided with the reduced abundance of *Ab. hystrix*. In the study presented here the two mite species that were less abundant on choked *El. repens*, viz. *T. urticae* and *Ac. mackenziei*, were also less abundant in general than *Ab. hystrix*, regardless of the presence of *Ep. bromicola* (Fig. 2a). This would be expected if the presence of *Ep. bromicola* in only a subset of *El. repens* plants provided a competitive advantage to local populations of *Ab. hystrix*, compared to *T. uriticae* and *Ac. mackenziei*, where all three species are utilizing *El. repens*. Manipulative experiments testing one mite species at a time will be necessary to properly quantify these interactions although such studies are complicated by the unpredictable nature of the development of the sexually reproductive forms of *Epichloë* spp. (characterized by the choke disease of their hosts), which may depend on environmental, geographical, or genotypic (either host or endophyte) factors (reviewed by Tadych et al. 2014). As such, although the results of the study presented here are preliminary, they provide information that should stimulate further investigation into the possible roles of endophytes in mite-plant interactions, which are largely unknown to date.

The results of this study suggest that herbivorous mite presence may be either positively or negatively correlated with the presence of choke in a grass host, depending on the species. This echoes studies of herbivorous mites sharing host plants with phytopathogenic fungi, in which either greater or lesser mite abundance has been observed in the presence of a fungus depending on the system. For example, mango bud tissue colonized by *Fusarium mangiferae* Britz, Wingfield & Marasas, and wheat and quackgrass leaves colonized by *Puccinia* spp., supported significantly higher populations of eriophyoid mites compared to healthy plants (Gamliel-Atinsky et al. 2010). Similarly, densities and incidence of *T. urticae* were greater on apple and cherry leaves infected with powdery mildew than on healthy leaves collected from orchards (Reding et al. 2001). Conversely, populations of *T. urticae* grew less rapidly on plants that had been inoculated with the fungal pathogen *Verticilium dabliae* Kleb. than on disease-free control plants (Karban et al. 1987). Herbivorous mites may increase the incidence and severity of fungal infection on host plants either by vectoring pathogen spores on their bodies (Batra and Stavely 1994; Abdel-Sater and Eraky 2001; Gamliel-Atinsky et al. 2010) or by providing wound-sites for fungal penetration (Petty et al. 2002; Cardenas et al. 2003). Of the mite species observed in this study, only *Ab. hystrix* appears to be a candidate for vectoring *Ep. bromicola* between *El. repens* plants, given the positive correlation between the presence of choke disease and *Ab. hystrix* abundance. Further studies would be required to ascertain such a role for this mite species.

As part of this study, the control plants (i.e. those without choke symptoms) were tested to ensure that asymptomatic *Ep. bromicola* was not present. However, given that many *Epichloë* spp., including *Ep. bromicola*, occur as either asexual, asymptomatic or sexual, choke-producing forms (Brem and Leuchtmann 2003; Schardl et al. 2013), one question that arises from this study is whether the observed differences in mite abundance in the presence of choke symptoms would also occur in the presence of the asexual, asymptomatic form of *Ep. bromicola*. Indeed, in one such experiment, fall armyworm (*Spodoptera frugiperda* Smith) larvae that were fed red fescue (*Festuca rubra* L.) infected with asymptomatic *Epichloë typhina* (Pers.) Tul., did not survive to pupation, compared to 43 % survival on red fescue without *Ep. typhina* (Clay et al. 1993), showing that the presence of choke-disease symptoms was not necessary to affect these herbivores.

Recent studies (Gange et al. 2007; Eschen et al. 2010) have revealed differing effects on insect herbivores from either single or multiple endophyte. It is not known if any additional endophyte species were present within the choked *El. repens* plants in this study, although given the breadth of endophyte species recorded from *Elymus* (Ringelberg et al. 2012) and other grass species (Baynes et al. 2012), it is possible that one or more asymptomatic endophyte species could have been present in combination with *Ep. bromicola* in the *El. repens* plants analyzed in this study, with unpredictable effects. Clearly, at this time the multitrophic effects of microbial plant symbionts represent a great opportunity for future study in the field of plant ecology.
